# The Political Economy of Health Inequality

**DOI:** 10.1177/27551938251365072

**Published:** 2025-08-11

**Authors:** Gary Lowery

**Affiliations:** ^1^School of Dentistry, 4591 University of Liverpool, Liverpool, UK

**Keywords:** political economy, health inequality, inequality, neoliberalism, government

## Abstract

How are health inequalities shaped by a country's political economy? In answering this question this article takes as its point of analytical departure health inequalities in England that are persistent, entrenched, and, by some metrics, increasing. Political economy in the English context is understood as broad commitment to neoliberalism as a governing paradigm. Partial answers to this question have already been provided through analyses of neoliberalism broadly conceived, as well as the impact of its key policy tenets (privatization, liberalization, and deregulation) on access to health and health care. The key contribution of this article, however, is to take a step back to consider the contributory role of the broader philosophical underpinnings of neoliberalism, thereby providing fresh insights into the manner in which the appropriate role of government, individualism, and inequality shape government understandings of, and responses to, health inequalities. In doing, so the article contributes to a greater understanding of the frequently neglected structural, or “upstream,” determinants of health inequalities.

The preceding four decades have witnessed a growing number of academic and government reports on health inequalities, with each highlighting their severity, trajectories, and appropriate policy responses.^1,2^ In 2010 one of the most important reports on the state of health inequalities in England—the Marmot Review—found that efforts to address health inequality were stalling.^3^ A decade later “The Marmot Review 10 Years On” announced that in the intervening years, life expectancy had fallen in the most deprived communities outside London for women and in some regions for men; and that for men and women everywhere time spent in poor health was increasing. These inequalities were exacerbated by the COVID-19 pandemic and cost of living crisis. Nevertheless, health inequalities are not inevitable, nor is the gap fixed. Rather, their prevalence and severity are heavily influenced by political economy considerations. This article provides an overview of the various means by which neoliberalism—the dominant political–economic paradigm in England—exerts such an influence.

This is not the first time a study of neoliberalism and its impact on health and health inequalities has been undertaken. Rather, a growing literature has sought to explain how its policy tenets—privatization, deregulation, and liberalization—have influenced health and health outcomes. Diverse examples include neoliberalism broadly conceived,^4^ health care,^5^ obesity,^6^ nursing home privatization,^7^ and access to health care for people with disabilities.^8^ What is nevertheless missing from much of the existing literature, and the key contribution of this article, is a greater foregrounding of how macro political–economic ideas influence understandings of, and responses to, health inequalities; specifically, how the broader philosophical underpinnings of neoliberalism (the appropriate role of government, individualism, and inequality) shape health inequality outcomes. In doing so, the article addresses a gap in the existing political economy (of health) literature, which is in its infancy compared to, for example, labor and the environment, issues on which political economists have had far more to say.^9^

Developing a greater understanding of this relationship nonetheless matters because health inequalities take an unnecessary and unjust toll on the health and length of life of much of the population, are a good measure of social and economic progress, are linked to earlier deaths and lost years of healthy life and have intergenerational effects from traumatic experiences.^10,11^ They also incur enormous economic costs, which Marmot and colleagues estimate at over £62.5 billion pounds per year through productivity losses, lost taxes, higher welfare payments, and additional National Health Service (NHS) health care costs.^11^

Against this backdrop, this article proceeds as follows. The first section provides a general introduction to neoliberalism and health inequality. The second, third, and fourth sections provide more detailed overviews of the contributory roles of the appropriate role of government, individualism, and inequality. The last section concludes with some broader reflections.

## Background

Political economy foregrounds the interrelationship between politics and economics and the impact on, for example, production, labor, and consumption. Notwithstanding the diversity of political–economic systems, this article is centrally concerned with neoliberalism, which has resided at the apex of ideational and policy practice in England for more than forty years, often advocated, if not wholly accepted, as common sense.^12–14^ The political economy of health can therefore be read as an attempt to understand the ways in which macro level structures (neoliberalism) shape individual and population health (and health inequality) outcomes.

As a concept neoliberalism has been critiqued for being both controversial and incoherent.^15^ Although this criticism is not dissimilar to the contested nature of other social science concepts (power, democracy, etc.), it nonetheless serves as a cursory reminder of the importance of conceptual clarity. For the purposes of this article neoliberalism is therefore understood as a phase of capitalism centrally concerned with the restoration of the wealth of the upper classes, achieved through a set of economic policies including liberalization, privatization, and deregulation, and rhetorically justified through claims to increased freedom, choice, and competition.

### Conceptualizing the Neoliberal Turn

The neoliberal turn in England can be conceptualized as a shift from “embedded” to “dis-embedded” liberalism.^16^ The context for this paradigm shift was the inflation and growth crises of the 1970s, which were discursively constructed as attributable to a set of Keynesian economic ideas that had resulted in “stop–go” cycles of growth and an excessively powerful state bureaucracy. Key to this crisis diagnostic was an increasingly powerful coalition of right-leaning actors. Against the backdrop of declining profitability in the 1960s–1970s, the material resources of businesses were mobilized to abolish tripartism (working relationships between workers, business, and government) in favor of a bilateral relationship between business and government. Discursive resources were also mobilized by, among others, the Mont Pelerin Society, a group of libertarians (including Milton Friedman and Friedrich Hayek) who strongly opposed the expansion of government and the welfare state. Throughout the 1970s these ideas were increasingly backed by major political figures and disseminated to the public through free-market think-tanks who provided policy briefs for distribution through the media.

Following the rejection of Keynesianism with the election of Margaret Thatcher in 1979, the government vigorously pursued an accumulation strategy that marked a significant departure from the goals, policies, and tools of embedded liberalism. This “finance-led” growth model—underpinned by reliance on consumption and debt-fueled growth—was evidenced by the “big bang” of financial deregulation and significant tax cuts for businesses and wealthy individuals on the one hand and tackling the abuse of welfare and inefficiencies of public provision on the other.^17^ In doing so, England became a pioneer of inflation targeting, privatization, deregulation, and liberalization. Government-controlled industries were privatized (telecommunications, energy, and railways), and services traditionally provided by the state outsourced (police custody, job training, fitness to work), the assumption being that the market would supply goods at lower cost and higher quality than the state.^17^

The corollary of a more circumscribed role for government was not, however, weak government. Rather the powers of the state were required to police the market and confront reform resistant groups. Nowhere was this more apparent than the Thatcher government's victory in the miner's strikes of 1984–1985 that both demonstrated its willingness to deploy the full force of the state and disciplined the organized working classes through forced deindustrialization, soaring unemployment, and reduced real wages.

Poverty and inequality increased but it was argued that this was relative, and that a rising tide of prosperity would lift all boats. Reality proved the converse. Aggregate gains and successful inflation targeting were accompanied by enormous distributional effects. A near trebling of the income share of the top one percent between 1979 and 2022 was accompanied by average annual income growth of −0.33 percent between 1979 and 1997 (and less than 1% thereafter) for the bottom fifth percentile.^18^ Poverty duly increased from 14 percent in 1979 to 22 percent in 2021–2022, including six million people in very deep poverty and 3.8 million people who experienced destitution.^19^ This rise is captured by the increasing numbers of people incapable of satisfying their basic needs, evidenced by the explosion of prefixes associated with poverty including food-, fuel-, pensioner-, and period-. Inequality likewise increased and in many instances became entrenched to the detriment of economic activity. Examples include opportunity,^20^ education,^21^ mental ill-health,^10^ dental health,^22^ and most pertinently for my purposes here, health.

Health inequalities relate to differences in the status of a population's health, differences in the care they receive, and their opportunities to lead healthy lives. The most cited metric is life expectancy, although health inequalities also include the availability of treatments, quality of care, and the wider determinants of health.^23^ These are influenced by and frequently analyzed across four factors: socioeconomic, geographical, specific characteristics, and socially excluded groups. Individuals can experience each or a combination of some or all these factors.

Health inequalities in England are significant, persistent, and in some instances, increasing. Prior to the COVID-19 pandemic, the gap in life expectancy between the most and least deprived areas in England had widened to 10.3 years for men and 8.3 years for women.^10^ This is lengthened when consideration is given to “healthy life expectancy” (time spent in good or very good health) and “disability-free life expectancy” (time spent without conditions or illnesses that limit people's ability to carry out daily activities). In 2015–2017 females in the area with the lowest healthy life expectancy (53.5 years) had a life expectancy 18.1 years lower than those in the area with the highest expectancy (71.6 years). At 15.1 years broadly similar inequalities exist for males (54.7 years versus 69.8 years).^10^

Health inequalities exist in some form in all countries. The United Kingdom is certainly not alone in this regard. Yet of all member countries of the Organisation for Economic Co-operation and Development (OECD), evidence suggests that they are particularly acute in the Anglo-liberal world, most notably in the United Kingdom and United States. Put simply, political economy considerations matter. The question is, how? In answer to this question, it is to the more specific philosophical underpinnings of neoliberalism that this article turns.

## Appropriate Role of Government

For neoliberals, the free market is an arena in which both parties can benefit from an exchange so long as cooperation is voluntary. Absent coercion, free choice means that if one person refuses to satisfy our wishes, we can turn to another.^24,25^ Resultantly, neoliberals broadly concur that the role of government should be limited to: (*a*) protecting society from external threats; (*b*) establishing an administration of justice; (*c*) providing public goods; and (*d*) protecting members of a society who cannot be considered “responsible”.^26^ Beyond these functions Friedman quotes Dicey in cautioning that “state help kills self-help”.^24^^, p.201^ This basic understanding informs health inequalities in three key ways.

First, health inequalities are often lamented for being unfair and unjust.^27–29^ Neoliberals are nonetheless skeptical of the concepts of fairness and justice, which are frequently utilized to justify the pursuit of social goals typically presented as the “common good” or “general interest.” This skepticism stems from their indeterminate meaning (government does not possess the collective moral standards required to determine what the common good is), the fallacy that conscience can be collectivized,^30^ and the inability of such terms to provide a clear course of policy action.

This problem is particularly acute in respect that health inequalities exist on a social gradient in which, broadly speaking, each group further along the income deciles is healthier than those before. Health inequalities are therefore experienced by everyone, not just those at the very bottom and top. Yet it is unclear which—a combination or all—deciles that proponents of action would tackle, to what extent, and how. Examples include Marmot's suggestion that “the aim should be for everyone in society to have the good health and length of life of those at the top”^11^ and the NHS’ Core20plus5 initiative, which contrastingly pledged to concentrate efforts on the 20 percent of the population in the lowest deprivation quintile.

Additional complexity stems from the fact that health inequalities are shaped by many factors, including income, housing, and education. The “comprehensive, multifaceted”^23^ nature of health inequality means it is unclear whether fiscal constraints would permit policymakers to pursue the resolution of one, a combination of some, or all factors simultaneously. Calls to tackle health inequality are therefore typically couched in vague terms including the need for inclusive growth, social value approaches, and building community capability.^10,11^ Should government interventions require a clear balance of benefits over costs (as proposed by Freidman ^26^), it is unclear that this basic objective has been, or can be, successfully realized.

With comprehensive, centrally directed strategies notable by their absence, responsibility for tackling health inequalities has increasingly been delegated to local authorities. Of particular note was the *Health and Social Care Act* (2012) which acknowledged that, nationally, accountability to protect against certain threats to health (e.g., infectious disease) rests with the central government. The *Act* nonetheless placed on local authorities “the responsibility for improving the health of their local populations” given that “many of the wider determinants of health (including housing, economic development, transport) can be more easily impacted by local authorities”.^31^^, p. 1^ Yet the ability of local authorities to exercise their duty has been severely constrained by cuts to public health and local authority (including social care) budgets of 24 percent and almost 29 percent, respectively, since 2010.^32^ Most pertinently, not only has funding declined, it has also been allocated regressively with absolute cuts in the poorest places as much as six times larger than in the least deprived.^11^

Second, even in such circumstances that there may exist broad agreement on the presence of an undesirable distribution of health inequalities, for neoliberals, government intervention has significant and adverse third-party effects. That is, government attempts to rectify a situation may make matters worse because whatever strategies exist for ameliorating human evils, they will themselves have costs, some of which will come in the form of other social ills. Former Conservative Prime Minister David Cameron (2009), for example, suggested that many of the problems facing society are the result of bad government and bad policies that would be eradicated if government failure was eliminated and personal responsibility restored.

Many of these arguments (re)gained prominence in the aftermath of the Great Crash of 2008–2009. Despite being attributable to a laissez-faire approach to financial sector regulation and supervision, the surge in government debt driven by significant bank bailouts and declining tax receipts brought about by the accompanying recession were, paradoxically, ascribed to the fiscal profligacy of the over-extended state^33^ by the Conservative Party and much of the media. In doing so, the need for reductions in government expenditure and appeals to the virtue of the small state (juxtaposed with the “big society” ^34^ in which volunteers would run formerly provided public services), were advocated as a new form of economic common sense.^35^

Marginalized within this discussion on the deleterious consequences of government action, however, is the fact that the government is constantly engaged in making decisions that have a significant impact upon health inequality outcomes. Extensive evidence shows, for example, that large commercial enterprises actively shape the way health issues are interpreted, framed, and responded to. These are the “commercial determinants of health”,^36^ the private sector activities that affect an individual's health. Corporations (including fossil fuel, tobacco, and junk food) influence health policy and outcomes through lobbying and party donations, framing consumer choice/action as a matter of individual responsibility, attempting to discredit science and scientists, promoting self-regulation, partnering with government, and using the media to shape public opinion and attitudes. The result is to delay or weaken effective public policy interventions, thereby negatively impacting health inequality.^37,38^

The conflict between business, profits, and health equity is certainly not new.^38^ Yet under neoliberalism corporations are much more capable of realizing their interests.^39^ This is attributable to the relative decline in the number of small firms, simultaneous rise of oligopolistic transnational corporations, immense concentrations of corporate power,^14^ and aggressive practices aimed at undermining market competitiveness. These firms, including in health-related industries such as food, beverages, and pharmaceuticals, push for further liberalization and deregulation while contrastingly lobbying for, and receiving, significant government subsidies and tax-breaks. Yet by presenting a vision of consumer-as-king, rhetorical commitment to freedom, choice, and competition (neoliberalism) have largely removed agency—and moral accountability—from corporations. Politics has therefore become almost unnecessary in this scenario because if consumers want clean air, ethically produced food, and good conditions for workers, they simply need to purchase goods that reflect these values.^39^ Indeed, the fact that people purchase a product is taken as proof of its, and the consumer's, value(s).^12^

Finally, a key component of freedom from government intervention is the ability to buy from, and sell to, anybody else so long as transactions are undertaken voluntarily. Without this, the argument proceeds, we are faced with coercion—the “control of the environment or circumstances of a person by another”.^40^ This is not to suggest that individuals will exercise their freedom in a manner that everyone agrees with. Indeed, Mises's suggestion that the average man indulges in habits that result in undesired effects does not bestow upon governments the right to legislate against the ability of individuals to satisfy their wants in the marketplace as this would be akin to telling them they are no better than guileless suckers requiring a paternal (interventionist state) guardian.^41^

This approach assumes that all agents have access to the same information, whether that be about the benefits and harms caused by, for example, smoking, eating particular foods, or recreational activities such as gambling. Yet there exist clear asymmetries of power and information that interfere with the ability of individuals to make rational and informed choices that reflect their interests.^13^ Examples include denying, questioning, or falsifying the harms caused by smoking and gambling, and misleading labeling (or outright false claims) regarding the health benefits of certain foods.

Nonetheless, many politicians have opposed efforts to manipulate the harmful lifestyle and dietary habits of individuals. Examples include increasing tobacco taxes, the “sugar tax” levy on soft drinks, and minimum alcohol pricing units. Although the evidence for the (public) health benefits of these interventions is well-established,^42^ strong resistance has been provided by counterarguments that, in the words of former Conservative Prime Minister Boris Johnson, such interventions “go against our instincts”^43^ and interfere with the freedom of individuals to exercise their own discretion as consumers. These arguments found resonance in the Conservative Party's 2005 Action on Health manifesto, which stated, “We do not believe that food producers are to blame if people eat unhealthily, or that pubs are to blame if people drink or smoke. There we shall seek voluntary, not statutory solutions to public health problems”.^44^ These arguments have proven particularly powerful in circumstances whereby the freedom of individuals to choose their own course of action, irrespective how unsavory, do not interfere with the rights of other people to do the same.

The essential point for neoliberals is that freedom from government intervention or coercion cannot be real freedom if it is only granted when it is known in advance that its effects will be beneficial. That is, if freedom was granted only if its intended use was consistent with our interpretation of the most appropriate application, the case for it would essentially disappear. Hayek therefore notes that it is important that freedom is extended to individuals and usage that to some seem undesirable.^40^ Indeed, some individuals may make poor choices with regards to their freedom and their health, and these may have adverse consequences for a country's health and health inequalities. Yet the question justly requiring consideration is, does it logically follow that government has a right to intervene to restrict freedom and coerce individuals into living healthier lives? The answer, to borrow from Sowell, is that because government can never know each individual's circumstance nor what they desire, the best it can do is respect the dignity of the individual and trust that they are exercising their freedom in a manner they see fit, irrespective of whether others believe this to be good or bad.^45^

## Individualism

Individualism formed the backbone of the neoliberal revival.^17^ Referred to by Hayek as the “antirationalist” approach at base is an assumption that individuals are not highly rational, but irrational, lazy, and fallible beings whose individual errors are corrected in the course of a social process.^46^ The aim of society ought therefore be to make the best of what is an imperfect material, as a truly free system is one in which freedom is granted to all, not just the good and the wise. The corollary of individualism, however, is not anarchism. What those subscribing to individualism do is simply seek to limit coercive power to fields where it is necessary to prevent coercion by others (the function provided by government). This implies the need for individuals to be free to pursue their own interests/aims (without which according to former Conservative Prime Minister John Major the state assumes such responsibility as to become “the keeper of the individual's conscience and duty” ^47^); and if an individual is free to choose their own course of action, they should also bear the risk attached to that choice. This understanding has three consequences for policy.

First, neoliberals foreground the boundless variety of human nature,^30^ and in doing so, suggest that individuals ought to be allowed to pursue what they think is desirable, the assumption being that the state cannot know or second-guess other people's wants, interests, values, or goals. Rather all are pursuing their interests, as they see them, guided by their own values,^26^ such that it would be immoral to take from them the right to make personal decisions.^47^ Resultantly, individuals ought to be left, within defined limits, to follow their own values and preferences rather than somebody else's. Indeed, it is this recognition that “individuals must be able to make up their own minds for themselves as to the kind of lives which they wish to live”^30^^, p.15^ that constitutes the base of the individualist position.

A commitment to individualism, however, is not naturally correlated with state inaction. Smoking is one notable example. Historically, the framing of cigarettes as a symbol of masculinity, female emancipation, and freedom of choice^48^ have cast a long shadow over public policy, with successive governments reluctant to introduce an outright ban on cigarette sales, despite smoking contributing to approximately half of all differences in health inequalities between the richest and poorest members of society.^49^ Rather, eschewing the most controversial “upstream” interventions, emphasis has been placed upon self-regulation, taxation as a means of reducing tobacco use, awareness raising, local incentives, treatment, and individual responsibility.

With strong lobbying from the medical industry and a shift in public opinion in the late 1990s, however, smoking indoors in public spaces was banned in England in 2007 by the Labour government. Although the public health rationale was stark, the fact that in 2005 11,000 deaths were attributable to passive smoking highlighted its dangerous third-party effects.^44^ Despite many Conservative MPs voting against the ban, third-party effect arguments were also mobilized in 2015 when the Conservative Government made it illegal to smoke in a car with passengers under the age of 18 present. Considered alongside commitment to the generational endgame ban, significant anti-smoking regulation serves as a cursory reminder that a commitment to individualism is not incompatible with substantive public health legislation, particularly in circumstances in which one individual's right to satisfy their wishes negatively impacts others’ right to do the same.

Second, notions of individual responsibility continue to predominate public debates as a corollary of individual choice.^48^ Thatcher's assertion that “the quality of our lives will depend upon how much each of us is prepared to take responsibility for ourselves”^50^^, p.29^ followed Friedman's contention that contrary to government or businesses, only people can have responsibilities.^51^ The idea is that freedom not only means that individuals have the *opportunity* to pursue their choices, but that they should also carry the *burden* of that choice. Freedom and responsibility are therefore conflated under the postulate that a free society cannot adequately function unless all participants agree that each individual occupies the position that results from their own actions. This assumption stems from the proposition that because everyone is given the opportunity to make use of the circumstances that only they themselves are aware of, we can only deduce that the outcome of an individual's position in the hierarchy of health inequalities is determined solely by them, unless the contrary is manifestly obvious. Examples include “infants, idiots, or the insane”,^40^ individuals who have either not learned enough or are incapable of learning. Yet the assumption is that so long as individuals are aware that they will be held responsible for their actions, they need to be treated as responsible. Consequently, being a free member of a community carries a burden as well as a privilege.

Emphasis on the simple maxim that “we are all responsible for our own actions”^52^ has had two important effects. Firstly, in the rare instances national health inequality reduction strategies are considered, these seldom, if ever, contain more than a cursory reference to their political/political economy “upstream”/macro-level determinants.^27^ Secondly, emphasizing individual responsibility has provided the ideological grounding for assaults on public services, welfare, and the education system.^15^ In the process, notions of public versus private goods have been fundamentally transformed as education (tuition fees), welfare (pensions), and health and social care provision (use of private providers) are increasingly individualized, considered an investment in an individual's future, and treated as goods to be traded in the marketplace. The result is to transfer responsibility for well-being back to the individual, inadequately capturing the consequences of the unequal distribution of resources including money, knowledge and political power,^53^ thereby leaving large sections of the population exposed to impoverishment and a poor range of health choices.^14^

Finally, it is broadly acknowledged within Public Health that people's behavior is a major determinant of how healthy they are.^23^ Public Health England's PHE Strategy 2020–2025, for example, suggests that four individual behaviors stand out, including “whether we choose to smoke, what and how much we choose to eat, whether we exercise and how much alcohol we drink”.^54^ Resultantly, policy interventions have typically been targeted at the individual, an approach that (re)gained prominence from 2010 with David Cameron's rejection of top-heavy, controlling government that sapped individual responsibility, to policies that emphasized incentives and greater provision of information.

The consequence of the individualist approach, however, is to downplay the significance of the broader context within which people act. That is, individuals inhabit an environment in which agency is bounded, institutional logics are difficult to escape, identities and interests are socially derived, and constraints on action (material and otherwise) restrict people's ability to lead healthy lives.^53^ The individualist approach therefore marginalizes robust evidence that health-related behaviors are enormously influenced by the “wider”—or “social”—determinants of health. These are the nonmedical social, economic, and environmental influences that impact people's health, including income and social protection, education, unemployment, working conditions, housing, early childhood development, and access to affordable health services of decent quality. Indeed, variation in the wider determinants of health is increasingly recognized as the fundamental cause (the “causes of causes”) of health outcomes, accounting for 30–55 percent of variation in health outcomes.^55^

Nonetheless, government policy has often worsened the wider determinants of health. On the one hand, social housing stock has declined significantly since 1979, average house prices have increased considerably, private renting has grown (21% of which stock failed to meet the decent homes standard in 2021), and homelessness has soared (notably since 2010).^56,57^ This trend has been driven by government deregulation, including the 1980 *Housing Act* (Right to Buy local authority homes at vastly discounted prices), huge cuts to council housing expenditure, the 1988 *Housing Act* that ended local authorities’ statutory requirement to directly meet housing needs, and “voluntarily” transferring housing stock to housing associations.^58^

On the other hand, under neoliberalism regulation has been rolled back in some areas to the benefit of businesses and rolled forward in others as individuals are increasingly managed and policed by state agencies, in an archetype example of Gamble's free market—strong state.^17^ Despite secure employment being one of the most important determinants of physical and mental health,^57^ unemployment is frequently blamed on individual failure to adapt to the requirements of the market or accept a wage low enough. Contrastingly, successive governments have legislated for tougher conditions for unionization, workfare programs, and the use of welfare sanctions, all of which have important (negative) effects on wages, working conditions, and mental health.^9^ This has been exacerbated by the shift from direct to indirect taxation, and the withdrawal of the state from many areas of social provision, the upshot being that many people are increasingly marginalized, dispossessed, and disenfranchised.^13,14^

The wider determinants of health therefore persist through changes in disease patterns, health care accessibility, genetics, and individual behavior, a point acknowledged by former Conservative Secretary of State for Health and Social Care Sajid Javid who noted that “we can’t tackle health disparities without tackling wider disparities too”.^59^ Nonetheless, beginning in 2010 the Coalition government aggressively pursued a program of austerity—reduction of real wages, prices, and public spending aimed at cutting the government deficit and debt.^60^ Policies included the tightest squeeze in the NHS real-term budget since the 1950s, massive cuts to local authority social care provision, intensified use of the Work Capability Assessment, a cap on total household benefits, and real-term benefit and tax credit cuts for three years (2013–2016).

Austerity increased wider, and health, inequities through both a “social risk effect” (increasing child poverty, soaring homelessness, a 51-fold increase in the number of food bank parcels distributed in the UK)^61–63^ and “health care effect” (cuts to health care services and restricted access to care).^64^ Moreover, mortality rates stopped improving in the early 2010s, increased in some of the most deprived areas, and contributed to approximately 335,000 more deaths between 2012 and 2019 than expected based on previous trends.^63,65^ Life expectancy likewise slowed, and in some cases reduced, for the first time in decades with some of the most deprived areas disproportionately impacted.^11,63^

## Inequality

On inequality, Hayek,^40^ Friedman,^24^ and Sowell^45^ all contend that irrespective of the type of society one inhabits (planned or market-driven), inequalities will always exist in some form, including in countries with highly developed welfare systems. Inequality is therefore taken as natural and attributed to individuals’ differing capacity for learning, making use of knowledge, and developing skills the market is willing to purchase. It follows logically that if people are treated equally, the result is inequality in their actual position. At the heart of neoliberalism therefore lays a tension between equality before the law and the desire to make people more alike, which is intolerable in a free society and likened to a new form of servitude.^46^ Three implications follow these premises.

First, neoliberals foreground the frequent failure to acknowledge the role of the individual personality in fostering inequalities. Indeed, the fact that we are, according to Thatcher, “very different in our skills, temperament, capacity for decision, and capacity for courage” ^30^^, p.15^ means that an individual attempting to become an actor or actress rather than civil servant makes a choice to enter a lottery. If both were paid equally, their incomes in a fundamental sense would be unequal. So actual inequality may be down to arrangements designed to satisfy individual's tastes.^24^

A similar logic applies to those electing to engage in activities that might adversely affect their health, whether that be smoking, using recreational drugs, consuming alcohol, not regularly engaging in physical activity, and consuming food and beverages high in fat and sugar. At the heart of neoliberalism, however, is belief in the dignity of the individual and their freedom to make the most of their leisure time and lifestyle habits according to their own lights, provided they do not interfere with the rights of other individuals to do the same.

This argument recognizes that individuals are very different, and indeed, in great measure rests on this assumption.^40,41^ It therefore follows that if individuals are assured of equal positions in life (including in the hierarchy of health inequalities), or if governments attempt to reduce unintended but inevitable differences in wealth and health, the result must be inequality in their actual position.^66^ An important distinction is therefore made between equality before the law and material equality. Given their irreconcilability, neoliberals advocate equality of the general rules of law as this is the only kind of equality conducive to liberty. This approach does not oppose health equality or greater health equality than presently exists, nor is it considered an undesirable condition. Indeed, both Friedman^26^ and Hayek^40^ concur that wherever government action is required, methods that incidentally reduce inequality may be preferable. It is simply to state that the desire to make people alike in their condition cannot be tolerated in a free society, which is opposed to discriminatory coercion to bring about a preconceived pattern of health distribution; that absent a clear burden of proof of policy effectiveness the solution ought, according to Thatcher, lay in the system's (neoliberal capitalism's) characteristics, including the market, families, friends, and moral traditions.^30^

Secondly, although inequality is a natural corollary of a market economy, emphasis is placed on its origins vis-à-vis alternative forms of societal organization. Hayek^40^ and Friedman,^24^ for example, both make a distinction between inequality in planned societies that is determined by a greater authority, characterized by rigidity year after year, more unequal, and symptomatic of a status society. This is contrasted with a market economy where inequality is attributed to the many circumstances that nobody could have foreseen.^24,41^ The assumption is that inequalities are not the consequence of design but are instead determined by impersonal market forces that generate social mobility and dynamic change such that the position of individuals and their families might reasonably be expected to vary from year to year.^24,66^ The market economy is therefore presented not only as desirable, but also efficient and ethical.^12^

These premises have important implications for policy (in)action to address (health) inequality, notably in relation to its short- and long-term dimensions. That is, per their critics, neoliberals broadly concur that in the short term the health of those in the lowest income decile could be improved through redistributive policies to fund welfare and increase public spending on health care and education. The counterargument, however, is that although potentially yielding short-term results, the consequence would be, in the long term, to prevent progress all the way down, thereby negatively affecting everyone. Indeed, Hayek suggests “there is no more effective way of making a society stationary than by imposing upon all something like the same average standard”.^40^^, p. 49^

A large body of evidence nonetheless highlights that since the 1980s the key contributors to health inequalities in England are both significant and entrenched. Numerous reports have noted, for example, that the poorest and the richest in the United Kingdom are the most socially immobile, which exacerbates social (and thereby health) inequalities as disadvantaged individuals are less likely to climb the income ladder—a scenario worsened significantly by the COVID-19 pandemic and cost of living crisis.^11,21,67^ This is exacerbated by the fact that the education system has failed to function as the great social leveler as children from poorer backgrounds do significantly, and progressively, worse at every stage of school.^21^ This matters because educational attainment is strongly linked with improved health behaviors and outcomes.^57^ Finally, babies from disadvantaged backgrounds are more likely to be born with low birth weight, which leads to worse health in childhood and worse outcomes in later life. Crucially, the soaring costs of housing and rising child poverty means that social mobility is predicted to worsen further in future years.^20^

A third, and final, point seldom acknowledged as explicitly as by Hayek is that “we may be free and yet miserable”,^40^^, p.18^ meaning that freedom does not mean all good things to all people, whether that be a life of “ease without effort”,^68^ nor the absence of evils, including the freedom to starve, live unhealthy lifestyles, or make costly mistakes. Indeed, freedom does not mean the freedom to choose from an infinite number of options, nor does it deny that opportunities to lead healthier lives are unequally distributed. Yet, Hayek suggests that “the question of how many courses of action are open to a person is, of course, very important. But it is a different question from that of how far in acting he can follow his own plans and intentions . . . directed toward ends for which he has been persistently striving rather than toward necessities created by others”.^40^^, p. 12^ The point is that it is better to have a choice between several potentially very unpleasant health choices than to be coerced by the government into one option.

Nevertheless, government policymaking has actively narrowed the potential choice options for individuals and exacerbated health inequalities in the process. This despite Theresa May's commitment to tackle the “burning injustices”^69^ of unequal life expectancy and Boris Johnson's flagship white paper that pledged “to tackle the stark disparities in health outcomes across the UK”.^70^ This has taken several forms. On the one hand, successive governments have taken little action to resolve the wider determinants of health, have favored downstream interventions aimed at prevention when they have, and have cut funding for all kinds of policy interventions. This matters, because as Lynch notes medical care is, counterintuitively, one of the less important drivers of ill health that is more likely to result from exposure, vulnerability, and treatment of health threats.^9^

On the other hand, England has some of the largest regional inequalities in Europe.^71^ Deprived areas are subject to a circular process in which as stocks of human, social, physical, and institutional capital evaporate, businesses and skilled individuals move out, thereby causing a vicious circle in which the quality and quantity of jobs and skills fall, as do measures of health, thereby locking individuals into a low growth, low income, low well-being equilibrium. For many people, relocating is not an option due to locked-in cycles of poverty, and a reluctance to move for nonpecuniary reasons such as family and community ties and attachments to local traditions.^72^ These areas, commonly referred to as “forgotten” or “ignored” communities, have been subjected to extended periods of economic decline, unemployment, and adverse public health outcomes including “deaths of despair” by alcoholism, drug abuse, and suicide.^73^

Finally, for individuals living in deprived areas, a lack of freedom and choice is exacerbated by inequitable access to crucial health services, with people in the poorest communities having fewer general practitioners per head of population and lower elective care admission rates despite having a higher disease burden. Resultantly, substantial differences exist in rates of avoidable mortality between geographical and population groups. Williams and colleagues, for example, note that in 2017 males in the most deprived areas were 4.5 times more likely to die from an avoidable cause than males in the least deprived areas, with the corresponding figure being 3.9 times for females.^23^

## Conclusion

The key argument of this article is that health inequalities are neither inevitable nor fixed but are heavily influenced by a country's political economy. This conclusion reflects more broadly on three related points. The first relates to the identification of a complex and multidimensional evidence-policy-politics (read here political–economy) gap identified by Lowery and colleagues,^74^ which suggests that evidence is only likely to translate into policy if it finds a receptive political (economy) environment. This helps to explain why a range of evidence-based policies such as more stringent taxes and restrictions on the purchase of alcohol and junk food are not implemented, despite being some of the most effective interventions for improving individual health and reducing health inequalities. The latter was exemplified by the release of a two-year report commissioned by the Department for Environment, Food and Rural Affairs that included recommendations to introduce a higher tax on salt and on sugar. Asked to comment on the day of publication, then Prime Minister Boris Johnson noted, “I’m not attracted to the idea of extra taxes on hard working people”.^75^ At one stroke, the fact that political–economy considerations had essentially eviscerated two years of evidence-based research serves as a cursory reminder that normative political–economy questions and considerations are seldom solved solely with evidence.

The other focus of this article has been on how neoliberalism has informed government perceptions of the problem of, and responses to, health inequality. Yet it is important to consider the extent to which advocacy of a small role for the state, individualism, and individual responsibility have permeated public consciousness. Research by the King's Fund, for example, has shown that a majority of the public support stronger government action on public health, including the soft drinks levy, minimum alcohol unit pricing and restricting junk food advertising. The research also suggests that while most people accept their responsibility to contribute to their own health and well-being, the most important influence is the economic, physical, and social environment in which they live. Support for so-called “nanny state” policies therefore illustrate the false dichotomy ascribed to either individual or state policies, as evidence suggests that people tend to have more nuanced views of the concept of responsibility, including broad support for regulation.

The third and final point is that neoliberalism is, to borrow from Thomas Sowell, one of two conflicting “visions” of how to organize a country's political economy. For contrary to a market driven economy an alternative vision identified by Sowell is one that exists to benefit others; has a high sense of moral duty; argues that institutions can be changed to provide solutions to a range of social evils including poverty, hunger and (health) inequality provided this is underpinned by sufficient moral commitment; favors state intervention as a means of addressing structural factors and constraints outside of individual control; makes use of a range of policy levers that have the potential to reduce health inequalities; and ultimately suggests that every approximation to the ideal should be preferred.^76^ Resultantly, each “vision” produces significantly divergent responses to the problem, policies, and political economies of health inequality.
